# TINCR: An lncRNA with dual functions in the carcinogenesis process

**DOI:** 10.1016/j.ncrna.2020.06.003

**Published:** 2020-07-09

**Authors:** Soudeh Ghafouri-Fard, Sepideh Dashti, Mohammad Taheri, Mir Davood Omrani

**Affiliations:** a Urology and Nephrology Research Center, Shahid Beheshti University of Medical Sciences, Tehran, Iran; b Genomics Research Center, Shahid Beheshti University of Medical Sciences, Tehran, Iran; c Urogenital Stem Cell Research Center, Shahid Beheshti University of Medical Sciences, Tehran, Iran

**Keywords:** lncRNA, Terminal differentiation-induced non-coding RNA, TINCR, Cancer

## Abstract

Long non-coding RNAs (lncRNAs) have prominent roles in the pathogenesis of human cancers. Several studies have shown oncogenic or tumor suppressor roles of lncRNAs in different human tissues. Thus, these transcripts have been regarded as putative targets in treatment of cancer. The lncRNA terminal differentiation-induced non-coding RNA (TINCR) has an especial position in this regard, as it exerts different opposite roles in the pathogenesis of different human cancers. While it is up-regulated in gastric, esophageal, bladder and breast cancer; it is down-regulated in glioma, retinoblastoma and prostate cancer. Notably, data regarding expression profile of this lncRNA in a number of human cancers such as colon cancer, squamous cell carcinoma, non-small cell lung cancer (NSCLC) and hepatocellular carcinoma (HCC) are controversial. Expression level of this lncRNA has been associated with clinical outcome in patients with gastric cancer, colorectal cancer, NSCLC and head and neck squamous cell carcinoma. Moreover, Kaplan-Meier analyses have shown correlation between expression levels of TINCR and patients survival in patients with lung cancer and HCC. A number of cellular pathways such as Wnt/β-catenin, ERK1/2‐SP3 and MAPK signaling pathways have been identified as targets of this lncRNA in different cancers. Moreover, the rs8113645, rs2288947 and rs8105637 within this lncRNA have been associated with risk of gastric and colorectal cancer. In conclusion, although the role of TINCR in the carcinogenesis is essential, based on the conflicting data regarding the direction of effect of this lncRNA, therapeutic targeting of this lncRNA is a complicated issue which should be considered in a tissue-specific or even individualized manner.

## 1 Introduction

Long non-coding RNAs (lncRNAs) as a group of transcripts with sizes more than 200 nt are considered as important regulators of genes expression and carcinogenesis process. These transcripts participate in multiple cellular processes such as epigenetic regulation of gene expression, modulation of expression at transcriptional and post-transcriptional levels, thus influencing cell proliferation, apoptosis, migration and stability of the genome [1]. Several lncRNAs have been demonstrated to affect the carcinogenesis process [2]. Notably, they usually exert either oncogenic roles or tumor suppressor role in human cancers [3]. Yet, a number of lncRNAs have recently identified that their role in the carcinogenesis process depends on the tissue where they are expressed. Among these lncRNAs is the terminal differentiation-induced non-coding RNA (TINCR) [[4], [5], [6], [7]]. This lncRNA has a 3733 nt length and its expression has been recognized at a late phase of human epidermal differentiation [8]. It regulates expression of several important differentiation genes such as *FLG*, *LOR*, *ALOXE3*, *ALOX12B*, *ABCA12*, *CASP14* and *ELOVL3* at post transcriptional level. Notably mutations in a number of these genes have been associated with skin diseases [8]. Functional studies have revealed the presence of a TINCR-a 25-nucleotide 'TINCR box' motif in target mRNAs that mediates interaction between these mRNAs and TINCR [8]. Additional *in silico* analyses predicted the role of TINCR in esophageal development as well [9]. Based on the data provided by Human Protein Atlas RNA-seq dataset, TINCR has been shown to have specific expression in skin, placenta and esophagus [9]. Subsequent studies reported its aberrant expression in a wide array of human malignancies. The most important note about this lncRNA is its different roles in the pathogenesis of different cancers. In this review, we summarize the available data regarding the role of this lncRNAs in human cancers.

## 2 Cell line studies

Several studies have assessed expression profile of this lncRNA in various cancer cell lines. These studies have also assessed he effects of silencing of or forced over-expression of this lncRNA in cell proliferation, apoptosis of invasive properties of these cells. TINCR silencing in human gastric cancer cell lines has decreased cell proliferation and colony formation [6]. In the following sections, we describe the role of this lncRNA in different cancer cell lines.

### 2.1 Gastric cancer

Expression of TINCR has been significantly higher in gastric cancer cell line compared with a human gastric epithelial cell line [10]. Notably, in gastric cancer cell lines, the nuclear transcription factor SP1 has a prominent role in induction of expression of this lncRNA. The oncogenic role of TINCR in gastric cancer cells is mediated through its interaction with STAU1 protein. This interaction can modulate stability and expression of KLF2 and subsequently regulate expression of cyclin-dependent kinase genes [6]. Moreover, E2F1 enhances expression of TINCR in gastric cancer cells. Forced over-expression of E2F1 enhances gastric cancer cells proliferation, while its silencing reduces cell proliferation through hindering cell cycle progression in these cells. This transcription factor enhances growth of gastric cancer cells via induction of TINCR expression. TINCR interacts with STAU1 protein to affect stability and expression of the CDKN2B transcript, thus enhancing the proliferation of these cells [11].

### 2.2 Colorectal cancer

In colorectal cancer cells, mechanistic studies revealed conflicting results. Small interfering RNA (siRNA)-mediated silencing of TINCR in HCT116 and HCT8 cells suppressed cell proliferation, inhibited colony forming capacity and decreased cells migration and invasion [12]. On the other hand, another study in SW620 and HTC116 cells demonstrated the role of this lncRNA in suppression of proliferation and migration. Yet, a radioresistant colorectal cancer cell line (SW620R) showed over-expression of TINCR. Notably, TINCR silencing decreased radioresistance of these cells [13].

### 2.3 Non-small cell lung cancer (NSCLC)

NSCLC is another cancer type in which mechanistic studies of TINCR showed conflicting results. This lncRNA has been shown to interact with BRAF to enhance its kinase activity, therefore resulting in activation of MAPK pathway [14]. On the other hand, other have shown that this lncRNA decreases expression of miR-21 in NSCLC and suppresses cancer cell migration and invasion [15].

### 2.4 Hepatocellular carcinoma (HCC)

In HCC, two distinct studies showed association between over-expression of this lncRNA and induction of apoptosis via regulation of P53 and enhancement of cell proliferation via modulation of the miR-214-5p/ROCK1 axis, respectively [16,17]. Consistent with the latter study, depletion of this lncRNA in other HCC cell liens decreased oncogenic behavior through modulation of miR‐218‐5p/DDX5/AKT signaling [18].

### 2.5 Breast cancer

SP1 has a similar role in induction of TINCR expression in breast cancer cell lines. Up-regulation of this lncRNA in these cells enhanced cell proliferation, anchorage-independent growth and inhibited cell apoptosis in these cells. Notably, TINCR exerts its oncogenic role through competing with miR-7 and modulating KLF4 expression [19]. Another study in breast cancer demonstrated the role of TINCR in stimulation of tumorigenesis through modulating expression of miR-125b and its target gene ERBB2. Through this molecular axis, TINCR inhibits apoptosis in breast cancer cells [20]. Finally, TINCR has been shown to be over-expressed in trastuzumab-resistant breast cancer cells compared with sensitive cells. Its silencing has changed the trastuzumab resistance phenotype and reversed the attained epithelial-mesenchymal transition (EMT) in these cells. Its interaction with miR-125b has been shown to release HER-2 and prompt trastuzumab resistance. The CREB-binding protein-mediated H3K27 acetylation at the promoter region of TINCR has been suggested as the underlying mechanism for over-expression of TINCR in breast cancer [21].

### 2.6 Other cancers

In esophageal squamous cell carcinoma cells (SCC), siRNA-mediated silencing of TINCR repressed cell proliferation, migration and invasion. Moreover, this approach led to induction of apoptosis and inhibition of cell cycle progression [22]. A single study in oral SCC verified the results of this study [23]. However, another study in diverse types of SCC originated from cervix, head and neck and lung implied a tumor suppressor role for this lncRNA as TINCR silencing by siRNA enhanced cell growth and migration of these cells [24].

Other studies in glioma [25], prostate cancer [5] and retinoblastoma [26] indicated a tumor suppressor role for TINCR. Table 1 summarizes the results of cell line studies that assessed expression and function of TINCR in various malignancies.

**Table 1:** Summarized results of studies which assessed expression of TINCR in cell lines (Δ: knock-down, Human gastric epithelial cell line (GES-1), Human normal esophageal epithelial cell line (HEEC), Human normal oral epithelial keratinocytes (HOK), The human bronchial epithelial cells (HBE), human normal breast epithelial cell line (MCF-10A), Normal human prostate epithelial cell lines (RWPE-1 and P69), SV-40-immortalized human uroepithelial cell line (SV-HUC-1), Human normal liver epithelial cell line (THLE‐2)).

Cancer type	Targets/Regulators and Signaling Pathways	Assessed cell lines	Function	Reference
Gastric cancer	STAU1, KLF2, CDKN2B/P15 and CDKN1A/P21	MGC803, BGC823, MKN45 and SGC7901, and the normal gastric epithelium cell line GES1	Δ TINCR: ↓ cell proliferation, ↓ colony formation, ↓ tumorigenicity, ↑ apoptosis ↑ TINCR: ↑ cell growth, ↑ cell cycle progression	[6]
	E2F1/TINCR/STAU1/CDKN2B signaling axis	MGC803, BGC823, MKN45, AGS, SGC7901 and GES-1	Δ TINCR: ↓ cell proliferation	[11]
	–	HGC27, AGS, SGC-7901, MGC803 and GES-1	An approximately two-fold upregulated expression could be observed in GC cell lines for the lncRNAs TINCR.	[10]
	miR-375/PDK1	KATO III, NCI-N87, HGC-27, and SNU-1	Δ TINCR: ↑ apoptosis, ↓ cell proliferation	[4]
Colorectal cancer	miR‐7‐5p/PI3K/Akt/mTOR signaling pathway	HCT116, HCT8, HT29, SW620 and SW480	↑ TINCR: ↑ proliferation, ↑ migration, ↑ invasion	[12]
	Wnt/β-catenin pathway	LoVo, RKO, SW620, HCT116, SW480 and LS174T	Δ TINCR: ↑ proliferation, ↑ migration, ↑ invasion	[7]
	–	SW620 and HTC116 cells	↑ TINCR: ↓ proliferation, ↓ migration	[13]
Esophageal squamous cell carcinoma (ESCC)	–	TE-13, KYSE-410, ECA-109, TE-1 and HEEC	Δ TINCR: ↓ proliferation, ↓ migration, ↓ invasion, ↓ progression of cell cycle, ↑ apoptosis	[22]
Squamous cell carcinoma (SCC)	ZNF750	93UV147, UMSCC1, CaSki	Δ TINCR: ↑ cell growth, ↑ migration, ↓ activation of differentiation-related gene	[24]
Oral squamous cell carcinoma (OSCC)	Wnt/β-catenin signaling pathway	SCC-9, CAL-27, HOK	Δ TINCR: ↓ proliferation, ↓ migration, ↓ invasion	[23]
Cutaneous squamous cell carcinoma (CSCC)	ERK1/2‐SP3 pathway	A431	Δ TINCR: ↓ apoptosis, ↓ autophagy	[27]
Non-small cell lung cancer (NSCLC)	MAPK signaling pathway	MRC-5, PC9, H1299, H522, A549, 95D, 95C, H2172 and 293T	Δ TINCR: ↓ migration, ↓ viability	[14]
	miR-29b	A549, H1299, H1650 and the human large cell lung cancer cells H460	Δ TINCR: ↓ proliferation	[28]
	miR-21	H650 and H1581	↑ TINCR: ↓ migration, ↓ invasion	[15]
Lung cancer	miR-544a/FBXW7	A549, H322, H460, GLC-82, SPC-A1 and HBE	↑ TINCR: ↓ proliferation, ↓ invasion	[29]
Hepatocellular carcinoma (HCC)	miR-137/miR-133a	HUH7	TINCR expression was suppressed by miR-137/miR-133a and it may play an oncogenic role in HCC differentiation, invasion, and metastasis.	[30]
	p53	SNU-182 and SNU-398	↑ TINCR: ↑ apoptosis	[16]
	miR-214-5p/ROCK1	H1581 and SNU-475	↑ TINCR: ↑ proliferation	[18]
	miR‐218‐5p/DDX5/AKT signaling	PLC/PRF/5, Hep3B, Huh7, HCCLM3 and THLE‐2	Δ TINCR: ↓ proliferation, ↓ invasion, ↑ apoptosis	[18]
Glioma	RPL36/STAT1/CDK2	**U87MG and U251**	↑ TINCR: ↓ proliferation, ↑ G1/S cell cycle arrest	[25]
Breast cancer	–	MDA-MB-453 and MCF-7	Δ TINCR: ↓ proliferation, ↑ apoptosis, ↓ cell cycle progression	[20]
	miR-7/KLF4	MCF-10A, MDA-MB-231, MDA-MB-435, MDA-MB-453, MDA-MB-468 and MCF-7	Δ TINCR: ↓ migration, ↓ invasion	[19]
	miR-125b, HER-2 and Snail-1	Trastuzumab-resistant SKBR-3-TR and BT474-TR cell lines	Δ TINCR: ↓ Trastuzumab resistance, ↓ epithelial-mesenchymal transition	[21]
Prostate cancer	TRIP13	LNCaP, PC3, DU145, 22Rv1, RWPE-1 and P69	↑ TINCR: ↓ proliferation, ↓ migration, ↓ invasion	[5]
Bladder cancer	–	5637 cells, SW780, SV-HUC-1	Δ TINCR: ↓ cell growth, ↑ apoptosis	[31]
Retinoblastoma	PTEN	Y79 and WERI-Rb-1	↑ TINCR: ↑ apoptosis	[26]

## 3 *In vivo* studies

A number of studies have investigated the effects of TINCR silencing or over-expression in xenograft animal models. In gastric cancer, the results of in vivo studies were consistent with the proposed oncogenic role of TINCR from cell line studies since two independent studies showed decreased tumor growth following TINCR knock-down in xenograft animal models [4,6]. In colorectal cancer, two in vivo studies revealed inconsistent results. Zhang et al. have compared the tumor growth in BALB/c mice after subcutaneous injection of HCT116-shTINCR or HCT116-NC. Notably, they reported larger sizes and more rapid growth of tumors in sh-TINCR group. Furthermore, these tumors had higher ki-67 proliferation index, higher rate of metastatic foci in the lung and the liver compared with the control group. Thus, they suggested that TINCR silencing intensely stimulates tumor growth and metastasis [7]. On the other hand, Yu et al. have shown that stable knockdown of TINCR in HCT116 cells inhibits cancer cells growth and metastasis in BALB/c nude mice [12]. Two independent in vivo studies in breast cancer reached the similar results confirming the oncogenic role of TINCR in this kind of cancer [19,21]. Notably, the latter also verified the effects of this lncRNA in induction of Trastuzumab resistance [21]. Besides, TINCR knock-down has suppressed growth of NSCLC in xenograft model through modulating expression of miR-29b [28]. However, experiments in a mouse xenograft model which was established through subcutaneous injection of glioma cells have shown lower growth rate and tumor weight in TINCR overexpressing cells. Furthermore, protein levels of Ki‐67 and RPL36 were lower in TINCR‐overexpressing tumors. Thus, TINCR has been shown to suppress glioma growth in vivo [25]. Table 2 shows summary of studies which assessed function of TINCR in animal models.

**Table 2:** Summary of studies which assessed function of TINCR in animal models (Δ: knock down or deletion).

Cancer type	Animal models	Function and comments	Reference
Gastric cancer	Athymic (nu/nu) mouse models	Δ *TINCR*: ↓ tumorigenesis of GC cells, ↓ tumor weight and size	[6]
	Nude mice	Δ *TINCR*: ↓ Tumor growth	[4]
Colorectal cancer	Male BABL/c nude mice	Δ TINCR: ↓ CRC cells growth, ↓ metastasis	[12]
	BALB/c nude mice	Δ TINCR: ↑ Tumor growth, ↑ metastasis	[7]
Oral squamous cell carcinoma (OSCC)	male BALB/c nude mice	↑ TINCR: ↑ Tumor growth, ↑ metastasis	[23]
Non-small cell lung cancer (NSCLC)	Nude mice	Δ TINCR: ↓ Tumor growth, ↓ tumor weight	[14]
	Nude mice	Δ TINCR: ↓ Tumorigenesis	[28]
Hepatocellular carcinoma (HCC)	BALB/c nude mice	Δ TINCR: ↓ Tumor growth, ↓ tumor weight	[18]
Glioma	Female BALB/c nude mice	↑ TINCR: ↓ Tumor growth, ↓ tumor weight	[25]
Breast cancer	Female BALB/c nude mice	Δ TINCR: ↓ Tumor growth	[19]
	Male BALB/c nude mice	Δ TINCR: ↓ Trastuzumab resistance, ↓ metastasis	[21]

## 4 Human studies

Several studies have compared expression of TINCR between human tumor samples and non-cancerous samples from the same tissues. A number of studies have reported up-regulation of this lncRNA in gastric, esophageal, bladder and breast cancer tissues compared with the corresponding non-cancerous tissues [4,6,28,31]. However, other studies have demonstrated down-regulated of TINCR in glioma, retinoblastoma and prostate cancer [5,25,26]. Notably, data regarding expression profile of this lncRNA in a number of human cancers such as colon cancer, squamous cell carcinoma, non-small cell lung cancer and HCC are controversial [7,12,[14], [15], [16],^22^,^23^,^30^]. Expression level of this lncRNA has been associated with clinical outcome in patients with gastric cancer [6], colorectal cancer [12], head and neck SCC [24], non-small cell lung cancer [14] and some other cancers. Notably, Kaplan-Meier analyses have shown correlation between expression levels of this lncRNA and patients survival in both directions. This issue has been reported in patients with lung cancer [14,15] and HCC [18,30]. Table 3 summarizes the results of studies which reported aberrant expression of TINCR in clinical samples.

**Table 3:** Summary of studies reported expression of TINCR in clinical samples (Gastrointestinal stromal tumors (GIST), Head and neck Squamous cell carcinoma (HNSCC), Disease free survival (DFS), overall survival (OS)).

Cancer type	Numbers of clinical samples (tissues, serum, etc.)	Expression (Tumor vs. Normal)	Kaplan-Meier analysis	Univariate cox regression	Multivariate cox regression	Reference
Gastric cancer (GC)	80 primary GC tissue samples and paired adjacent non-tumor tissue samples	Up	The high TINCR patients had higher recurrence rates than the low-TINCR patients.	TINCR expression was a significant prognostic indicator of DFS in patients with GC	TINCR expression was a significant prognostic indicator of DFS in patients with GC	[6]
	Plasma samples from 162 patients with GC, 110 healthy controls, 28 patients with precancerous lesions and 21 with GIST	Up	–	–	–	[10]
	56 primary GC tissue samples and paired adjacent non-tumor tissue samples	Up	–	–	–	[4]
Colorectal cancer	80 pairs of CRC tissues and adjacent non-tumor tissues, 80 CRC plasma and 80 normal control	Up	High expression of TINCR predicted a poor OS (*p* < 0.001).	High expression of TINCR was predictors for poor OS.	High expression of TINCR was an independent predictor of poor OS.	[12]
	44 CRC tissues and their paired normal colorectal mucosa	Down	–	–	–	[7]
Esophageal squamous cell carcinoma (ESCC)	56 pairs of primary ESCC tissues and adjacent normal tissues	Up	–	–	–	[22]
Squamous cell carcinoma (SCC)	TCGA cohort of HNSCC patients	Down	Low levels of TINCR were significantly correlated with poor outcome of patients with HNSCC.	–	–	[24]
Oral squamous cell carcinoma (OSCC)	48 pairs of primary OSCC tissues and adjacent normal tissues	Up	Patients with high TINCR had reduced OS and progressive-free survival rates, compared with patients with low expression of this lncRNA.	–	–	[23]
Non-small cell lung cancer	98 pairs of NSCLC tissues and normal adjacent tissues	Up	High TINCR expression was correlated with poor survival.	–	–	[14]
	37 pairs of NSCLC tissues and normal adjacent tissues	Up	–	–	–	[28]
	70 pairs of NSCLC tissues and normal adjacent tissues	Down	Patients with low TINCR levels in NSCLC tissues had much worse survival rate.	–	–	[15]
Lung cancer	45 cases of lung cancer tumor tissues and adjacent normal tissues	Down	–	–	–	[29]
Hepatocellular carcinoma (HCC)	248 pairs of HCC tissues and normal adjacent tissues	Up	Patients with high TINCR expression tended to have worse DFS and OS.	–	TINCR was an independent poor prognostic indicator for DFS and OS in HCC.	[30]
	66 pairs of HCC tissues and normal adjacent tissues	Down	Low TINCR levels in HCC tissues were associated with low 5-year OS.	–	–	[16]
	60 pairs of HCC tissues and normal adjacent tissues	Up	–	–	–	[18]
	56 pairs of HCC tumor tissues and adjacent normal liver tissues	Up	High TINCR expression was correlated with worse OS.	–	–	[18]
Glioma	61 pairs of glioma tissues and normal adjacent tissues	Down	–	–	–	[25]
Breast cancer	26 GEO datasets with 4140 breast cancer including patients' long-term follow-up information	Up	High TINCR expression was correlated with worse OS.	–	–	[20]
	24 cases of breast cancer tumor tissues and adjacent normal tissues	Up	High TINCR expression was correlated with worse prognosis.			[19]
	60 patients with HER-2+ breast cancer who underwent surgical resection followed by trastuzumab treatment and primary cancer tissues before performing trastuzumab treatment	Up	High TINCR expression was associated with poor survival in patients receiving trastuzumab therapy.	–	–	[21]
Prostate cancer	52 pairs of normal prostatic tissues and prostate cancer tissues from TCGA database, Fresh 160 prostate cancer tissue specimens and 30 paired normal prostatic tissue specimens from 160 patients	Down	Level of TINCR is positively correlated with the OS of prostate cancer cases.	TINCR low expression was an unfavorable prognostic factor for prostate cancer patients.	–	[5]
Bladder cancer (BCa)	49 paired BCa tissues and corresponding noncancerous tissues	Up	–	–	–	[31]
Retinoblastoma (RB)	60 pairs of RB tissues and normal adjacent tissues	Down	–	–	–	[26]

Based on the importance of lncRNAs in differentiating the disease status in clinical samples, a number of studies have assessed the diagnostic power of TINCR in different cancer types. This approach has been performed on both tissue samples and plasma samples, Based on the measured area under curve (AUC) values in receiver operating characteristic (ROC) curves, the best diagnostic power has been reported in colorectal cancer where plasma levels of this lncRNA could differentiate patients from healthy controls with 92% accuracy [12]. Moreover, expression levels of this lncRNA could discriminate oral SCC tissues from adjacent non-cancerous tissues with diagnostic power of 87% [23]. Table 4 summarizes the results of studies which assessed diagnostic power of this lncRNA in clinical samples.

**Table 4:** Diagnostic value of TINCR in cancers.

Cancer Type	Numbers of clinical samples	Distinguish between	Area Under Curve	Sensitivity	Specificity	Reference
Gastric cancer (GC)	80 GC tissue samples and paired adjacent non-tumor tissue samples	GC tissue vs paired adjacent non-tumor tissue	0.701	0.65	0.71	[6]
	30 patients and 30 controls	Plasma TINCR levels from patients with GC vs. healthy controls.	0.70	0.73	0.63	[10]
Colorectal cancer	80 patients with CRC and 80 healthy controls	Plasma TINCR levels from patients with CRC vs. healthy controls.	0.922	97.5	80.0	[12]
Oral squamous cell carcinoma (OSCC)	48 paired OSCC and adjacent non-tumor tissues	OSCC tissue vs paired adjacent non-tumor tissue	0.871	75.0	85.4%	s [23]

TINCR has a number of single nucleotide polymorphisms (SNPs) which might affect expression or function of this lncRNA. Based on the importance of this lncRNA in human malignancies, these SNPs are expected to modify the risk of cancer. Ma et al. have assessed association between four tag SNPs (rs8113645, rs2288947, rs8105637, rs12610531) across the entire TINCR locus and risk of gastric cancer in a Chinese population [32]. Notably, they reported associations between rs8113645 and rs2288947 variant alleles and decreased risks of gastric cancer in the assessed population. Genotypes having A allele of rs8113645 and G allele of rs2288947 reduced risk of this cancer. Particularly, the observed associations were more prominent in younger persons, males, nonsmokers, and persons from rural areas. The functional role of the rs8113645 has been verified through the observed lower tissue expression of TINCR in GA + AA genotype carriers [32]. In another study, Zheng et al. assessed association between three tag SNPs (rs2288947, rs8105637 and rs12610531) and colorectal cancer in a Chinese population. They reported associations between rs2288947 and rs8105637 SNPs and risk of this cancer in their cohort of patients. Notably, the mentioned SNPs were cancer metastasis to lymph nodes as well [33]. Table 5 summarizes the results of studies which assessed association between TINCR SNPs and risk of cancer.

**Table 5:** TINCR polymorphisms and their association with cancer risk.

Cancer type	Cases/Control	SNP ID	OR (95%CI)	p-value	Description	Reference
Gastric cancer	602/602	rs8113645	0.70 (0.55–0.89)	0.003	GA and AA genotypes were significantly associated with decreased GC risk. GA + AA genotype carriers had lower TINCR mRNA expression levels compared with common genotype in both normal and GC tissues.	[32]
		rs2288947	0.83 (0.70–0.99)	0.037	AG and GG genotypes were significantly associated with decreased GC risk.	
Colorectal cancer	1400/1400 (Stage I: 600, Stage II: 800)	rs2288947	0.77 (0.67–0.88)	1.2 × 10^−4^	The G allele was associated with 23% decreased risk of colorectal cancer.	[33]
		rs8105637	1.22 (1.09–1.37)	6.2 × 10^−4^	The A allele was associated with 22% increased risk of colorectal cancer.	

## 5 Conclusions

Several lncRNAs have been reported to affect carcinogenesis process [3]. Among the newly assessed lncRNAs in this regard is the TINCR. TINCR is an lncRNA whose function in human malignancies is tissues-specific. This means that TINCR exert oncogenic effects in some human tissues and tumor suppressive effects in others. This observation can be explained by a tissue-specific influence of this lncRNA on distinct signaling pathways and molecular targets which are prominent in each tissue. However, a more complicated issue has been raised when assessing function of this lncRNAs in some tissues such as colon, lung and hepatic tissues in which different studies revealed controversial results regarding the role of this lncRNA. In cancer cell lines, this observation might be explained by the diversity of cancer cell liens, passage number and other culture conditions. Yet, when this issue is assessed in clinical samples, it best reflects the heterogeneity of cancerous cells and their behavior and necessitates a personalized approach for treatment of cancer patients. Perhaps, the presence of other genetic or environmental factors is important in determination of the role of this lncRNA.

TINCR has a sponging effect on a number miRNAs. For instance, in gastric tissues, it exerts a competing endogenous RNA (ceRNA) role to modulate PDK1 expression by sponging miR-375 [4]. In colorectal and hepatic tissues, TINCR has been shown to exert its effects through sponging miR-7-5p and miR-214-5p, respectively [12,18]. Finally, in lung tissues, it regulates cell proliferation through sponging miR-29b [28]. Thus, a possible explanation for tissue-specific role of this lncRNA might be the relative abundance of its targets in each tissue.

Although several studies have addressed the potential of lncRNA-specific strategies for cancer treatment [34], modalities that alter expression and function of TINCR should be applied with especial caution. A theophylline controllable RNA interference strategy has been successfully switches expression of this lncRNA in bladder cancer tissues [31]. Such controllable molecular approaches are promising methods in cancer biology. However, if these strategies are being tested in clinical settings, more strict criteria should be applied for selection of patients and cancer types.

In addition to the mechanistic studies which showed regulatory effects of TINCR on cancer-related pathways such as Wnt/β-catenin, ERK1/2‐SP3 and MAPK signaling pathways and important tumor suppressor genes such as P53, the presence of genomic variants within this lncRNA that influence risk of human cancer support the importance of TINCR in the carcinogenesis. Meanwhile, different roles of this lncRNA in diverse tissues further support the critical role of tissue context or tumor microenvironment in this process. Thus, a holistic point of view which is brought by a system biology method is required for understanding the complex interaction network that determines or modifies the role of TINCR in each tissue or context.

## Declaration of competing interest

The authors declare they have no conflict of interest.

## References

[1] Grixti J.M., Ayers D. (2020). Long noncoding RNAs and their link to cancer. Non-coding RNA Research.

[2] Youness R.A., Gad M.Z. (2019). Long non-coding RNAs: functional regulatory players in breast cancer. Non-coding RNA Res..

[3] Taheri M., Omrani M.D., Ghafouri-Fard S. (2018). Long non-coding RNA expression in bladder cancer. Biophys. Rev..

[4] Chen Z., Liu H., Yang H., Gao Y., Zhang G., Hu J. (2017). The long noncoding RNA, TINCR, functions as a competing endogenous RNA to regulate PDK1 expression by sponging miR-375 in gastric cancer. OncoTargets Ther..

[5] Dong L., Ding H., Li Y., Xue D., Liu Y. (2018). LncRNA TINCR is associated with clinical progression and serves as tumor suppressive role in prostate cancer. Cancer Manag. Res..

[6] Xu T., Liu X., Xia R., Yin L., Kong R., Chen W. (2015). SP1-induced upregulation of the long noncoding RNA TINCR regulates cell proliferation and apoptosis by affecting KLF2 mRNA stability in gastric cancer. Oncogene.

[7] Zhang Z-y, Lu Y-x, Zhang Z-y, Chang Y-y, Zheng L., Yuan L. (2016). Loss of TINCR expression promotes proliferation, metastasis through activating EpCAM cleavage in colorectal cancer. Oncotarget.

[8] Kretz M., Siprashvili Z., Chu C., Webster D.E., Zehnder A., Qu K. (2013 Jan 10). Control of somatic tissue differentiation by the long non-coding RNA TINCR. Nature.

[9] Iwakiri J., Terai G., Hamada M. (2017 Jun 8). Computational prediction of lncRNA-mRNA interactions by integrating tissue specificity in human transcriptome. Biol. Direct.

[10] Zhang K., Shi H., Xi H., Wu X., Cui J., Gao Y. (2017). Genome-wide lncRNA microarray profiling identifies novel circulating lncRNAs for detection of gastric cancer. Theranostics.

[11] Xu T.-P., Wang Y.-F., Xiong W.-L., Ma P., Wang W.-Y., Chen W.-M. (2017). E2F1 induces TINCR transcriptional activity and accelerates gastric cancer progression via activation of TINCR/STAU1/CDKN2B signaling axis. Cell Death Dis..

[12] Tao S., Wang L., Zhu Z., Liu Y., Wu L., Yuan C. (2019). Adverse effects of bisphenol A on Sertoli cell blood-testis barrier in rare minnow *Gobiocypris rarus*. Ecotoxicol. Environ. Saf..

[13] Kang Z., Jifu E., Guo K., Ma X., Zhang Y., Yu E. (2019). Knockdown of long non-coding RNA TINCR decreases radioresistance in colorectal cancer cells. Pathol. Res. Pract..

[14] Zhu Z.-J., He J.-K. (2018). TINCR facilitates non-small cell lung cancer progression through BRAF-activated MAPK pathway. Biochem. Biophys. Res. Commun..

[15] Xia H., Xiu M., Gao J., Jing H. (2019). LncRNA PLAC 2 downregulated miR-21 in non-small cell lung cancer and predicted survival. BMC Pulm. Med..

[16] Zheng Y., Lv P., Wang S., Cai Q., Zhang B., Huo F. (2019). LncRNA PLAC2 upregulates p53 to induce hepatocellular carcinoma cell apoptosis. Gene.

[17] Hu M., Han Y., Zhang Y., Zhou Y., Ye L. (2020). lncRNA TINCR sponges miR-214-5p to upregulate ROCK1 in hepatocellular carcinoma. BMC Med. Genet..

[18] Zhao H., Xie Z., Tang G., Wei S., Chen G. (2020). Knockdown of terminal differentiation induced ncRNA (TINCR) suppresses proliferation and invasion in hepatocellular carcinoma by targeting the miR‐218‐5p/DEAD‐box helicase 5 (DDX5) axis. J. Cell. Physiol..

[19] Liu Y., Du Y., Hu X., Zhao L., Xia W. (2018). Up-regulation of ceRNA TINCR by SP1 contributes to tumorigenesis in breast cancer. BMC Cancer.

[20] Xu S., Kong D., Chen Q., Ping Y., Pang D. (2017). Oncogenic long noncoding RNA landscape in breast cancer. Mol. Cancer.

[21] Dong H., Hu J., Zou K., Ye M., Chen Y., Wu C. (2019). Activation of LncRNA TINCR by H3K27 acetylation promotes Trastuzumab resistance and epithelial-mesenchymal transition by targeting MicroRNA-125b in breast Cancer. Mol. Cancer.

[22] Xu Y., Qiu M., Chen Y., Wang J., Xia W., Mao Q. (2016). Long noncoding RNA, tissue differentiation‐inducing nonprotein coding RNA is upregulated and promotes development of esophageal squamous cell carcinoma. Dis. Esophagus.

[23] Chen F., Qi S., Zhang X., Wu J., Yang X., Wang R. (2019). lncRNA PLAC2 activated by H3K27 acetylation promotes cell proliferation and invasion via the activation of Wnt/β-catenin pathway in oral squamous cell carcinoma. Int. J. Oncol..

[24] Hazawa M., Lin D., Handral H., Xu L., Chen Y., Jiang Y. (2017). ZNF750 is a lineage-specific tumour suppressor in squamous cell carcinoma. Oncogene.

[25] Hu Y.W., Kang C.M., Zhao J.J., Nie Y., Zheng L., Li H.X. (2018). Lnc RNA PLAC 2 down‐regulates RPL 36 expression and blocks cell cycle progression in glioma through a mechanism involving STAT 1. J. Cell Mol. Med..

[26] Song L., Qi Y., Lin M. (2020). Long noncoding RNA PLAC2 regulates PTEN in retinoblastoma and participates in the regulation of cancer cell apoptosis. Oncol. Lett..

[27] Zhou W., Zhang S., Li J., Li Z., Wang Y., Li X. (2019). lncRNA TINCR participates in ALA‐PDT‐induced apoptosis and autophagy in cutaneous squamous cell carcinoma. J. Cell. Biochem..

[28] Liu G., Yang H., Xu C. (2018). Long noncoding RNA TINCR promoted cell proliferation through sponging miR-29b in non-small cell lung cancer. Int. J. Clin. Exp. Med..

[29] Liu X., Ma J., Xu F., Li L. (2018). TINCR suppresses proliferation and invasion through regulating miR-544a/FBXW7 axis in lung cancer. Biomed. Pharmacother..

[30] Tian F., Xu J., Xue F., Guan E., Xu X. (2017). TINCR expression is associated with unfavorable prognosis in patients with hepatocellular carcinoma. Biosci. Rep..

[31] Chen Z., Liu Y., He A., Li J., Chen M., Zhan Y. (2016). Theophylline controllable RNAi-based genetic switches regulate expression of lncRNA TINCR and malignant phenotypes in bladder cancer cells. Sci. Rep..

[32] Ma X., Huang C., Luo D., Wang Y., Tang R., Huan X. (2016). Tag SNPs of long non-coding RNA TINCR affect the genetic susceptibility to gastric cancer in a Chinese population. Oncotarget.

[33] Zheng Y., Yang C., Tong S., Ding Y., Deng W., Song D. (2017). Genetic variation of long non-coding RNA TINCR contribute to the susceptibility and progression of colorectal cancer. Oncotarget.

[34] Jiang M.-C., Ni J.-J., Cui W.-Y., Wang B.-Y., Zhuo W. (2019). Emerging roles of lncRNA in cancer and therapeutic opportunities. Am. J. Cancer Res..

